# The droplet size of emulsion adjuvants has significant impact on their potency, due to differences in immune cell-recruitment and -activation

**DOI:** 10.1038/s41598-019-47885-z

**Published:** 2019-08-08

**Authors:** Ruchi R. Shah, Marianna Taccone, Elisabetta Monaci, Luis A. Brito, Alessandra Bonci, Derek T. O’Hagan, Mansoor M. Amiji, Anja Seubert

**Affiliations:** 1 GSK, Siena, Italy; 2 GSK, Cambridge, MA USA; 3 GSK, Rockville, MD USA; 4 Northeastern University, Boston, MA USA; 5 Present Address: Moderna Therapeutics, Cambridge, MA 02139 USA

**Keywords:** Adjuvants, Adaptive immunity

## Abstract

Self-emulsification is routinely used for oral delivery of lipophilic drugs *in vivo*, with the emulsion forming *in vivo*. We modified this technique to prepare novel oil-in-water emulsions of varying droplet size and composition on bench to enable adjuvanted vaccine delivery. We used these formulations to show that smaller droplets (20 nm) were much less effective as adjuvants for an influenza vaccine in mice than the emulsion droplet size of commercial influenza vaccine adjuvants (~160 nm). This was unexpected, given the many claims in the literature of the advantages of smaller particulates. We also undertook cell-recruitment mechanistic studies at site of injection and draining lymph nodes to directly address the question of why the smaller droplets were less effective. We discovered that emulsion droplet size and composition have a considerable impact on the ability to recruit immune cells to the injection site. We believe that further work is warranted to more extensively explore the question of whether, the smaller is not ‘better’, is a more common observation for particulate adjuvants.

## Introduction

Vaccine adjuvant research has traditionally been empirical and challenging, with new adjuvants typically taking at least a decade to advance from bench to clinic. Recent reviews on adjuvants by O’Hagan and Fox^1,2^ explained the history of adjuvant discovery and highlighted that the path forward would clearly benefit from using more rational design approaches, with a greater mechanistic understanding. Currently, a range of particulate adjuvants, including oil-in-water based emulsions are used as successful adjuvants in some settings. Emulsion adjuvants like MF59 and AS03 are included in commercially available influenza vaccines, and their composition and performance have been widely studied and published^3^. Droplet size of ~160 nm size in these adjuvants is determined by their production process of microfluidization.

Our work has been focused on trying to better understand how physical characteristics like size or droplet composition of emulsion adjuvants can impact their *in vivo* potency^4^. In a previous paper, we described a novel and simple process of self-emulsification, which enabled the generation of smaller emulsion droplet sizes than had previously been described. The novel Self-Emulsifying Adjuvant 20 (SEA20) was compared to the commercial benchmark MF59^4^. However, we observed that more potent immune responses were induced in mice with emulsions with a more typical droplet size of 160 nm, versus the newly created 20 nm droplets^5^. This is perhaps reassuring, since the established emulsion adjuvants (MF59 and AS03), both comprise droplets of ~160 nm size. Nevertheless, the literature over the last decade is replete with many suggestions of the superior potency of various nanoparticulates, although a review of this literature failed to convince us that the claims are fully substantiated^3^.

In the current work, we challenged the question of the impact of droplet size further, by creating self-emulsifying adjuvants of various sizes and evaluated them competitively in mice as an adjuvant for an established influenza vaccine. Moreover, once the previous observation of the enhanced potency of 160 nm droplets over 20 nm was confirmed, we used established mechanistic approaches to determine why this difference in potency was observed. Here, we describe the production of an alternative Self-Emulsifying Adjuvant 160 (SEA160), a squalene oil-in-water emulsion with size similar to MF59, and a comparable composition. In addition, we used SEA20 to evaluate the impact of droplet size on the adjuvant potency for a trivalent influenza vaccine (TIV). We observed that larger emulsion droplets (160 nm) resulted in better recruitment of immune cells to the site of injection (SOI) which in turn resulted in greater antigen uptake, faster translocation to draining lymph nodes (dLN) and improved cellular and humoral responses. We believe that we have made an interesting observation that droplet particle size matters for emulsion adjuvants, but contrary to what is often suggested in the literature, smaller is not better^3^. Further work is warranted to determine whether this is a common observation for particulate adjuvants or an exclusive one for squalene based emulsion adjuvants. Nevertheless, approaches will need to be established to allow the preparation of distinctively sized particulates of similar composition, like the SEA process described here, to allow this question to be more broadly addressed^3^.

## Materials and Methods

### Materials

Squalene oil, sorbitane trioleate (Span 85) and phosphate buffered saline (PBS) were obtained from Sigma Aldrich and polysorbate 80 (Tween 80) from Acros Organic. Millipore MilliQ deionized water was used and citrate buffer was acquired from Teknova. Chromatography column was obtained from Waters: Acquity UPLC (R) BEH C18 1.7 µm 2.1 × 50 mm.

### Formulations

#### Self-emulsification to create novel 160 nm-sized emulsion

Using the components of MF59, we studied different concentrations of excipients to obtain a ratio that can self-emulsify around 160 nm size, method described in^5^. In brief, self-emulsification was achieved by mixing specified amounts of oil and surfactant before introducing it to heated aqueous phase to achieve a crude oil-in-water emulsion. This emulsion was re-heated with gentle agitation to achieve a homogenous emulsion.

#### Physical characterization of SEA160

SEA160 was filtered through 0.22 µm membrane filter prior to characterization. Physical characterization of the emulsions was done for size, polydispersity index, pH, osmolality and squalene content; details in^5^. Size and zeta potential were measured on DLS with 1:100 dilution with deionized water. pH and osmolality were measured without diluting the adjuvant. Squalene content was determined by reverse phase liquid chromatography. SEA160 was analyzed on JEOL 100X transmission electron microscope (TEM) (Peabody, MA); 1% uranyl acetate was used for negative staining.

#### Reference emulsions

Emulsion adjuvants “Self-emulsifying adjuvant 20 (SEA20)”, “Microfluidized Adjuvant 90 (MFA90)” and “Microfluidized Adjuvant 160 (MFA160)” were formulated and used for *in vivo* comparison studies as previously described^5^. These microfluidized adjuvants are of the same composition as SEA20, but their droplet sizes are 90 nm and 160 nm respectively. The set of different emulsions together with the newly created “Self-emulsifying adjuvant 160 (SEA160)” and the benchmark MF59 allows the controlled comparison of emulsion formulations with the same composition and different size, as well as emulsions of same size and rising oil-content (Table 3).

### Mouse immunization

All animal studies were performed at Novartis Vaccines Research Centers in Siena or Cambridge, MA (in March 2015 the Novartis non-influenza Vaccines business was acquired by the GSK group of companies) in compliance with Institutional Guidelines and national laws. All animal protocols have been reviewed and approved by the respective Ethic Committees for Animal work.

### Assessment of humoral responses - ELISA and hemagglutinin inhibition (HI) titers

Six to eight weeks old female Balb/C mice from Charles River were immunized twice and bled prior to immunization, 3 weeks after 1^st^ immunization (3wp1) and 2 weeks post 2^nd^ immunization (2wp2). Equal amounts of trivalent inactivated influenza vaccine (TIV) antigens were used in the *in vivo* studies: H1N1 A/California/7/09, H3N2 A/Texas/50/2012 and B/Massachusetts/2/2012 at 0.1 µg or 1 µg dose each per animal. Vaccine groups were: untreated group injected with PBS alone (PBS), non-adjuvanted TIV (TIV), and TIV adjuvanted with SEA20, MFA90, MFA160, SEA160, diluted SEA160 and diluted MF59. Diluted SEA160 and diluted MF59 had equivalent squalene concentration to SEA20, MFA90 and MFA160 of 1.49% oil volume/total volume (volume/volume; v/v). Antigen and adjuvant solutions were mixed in a 1:1 ratio v/v at 0.1 µg and 1 µg TIV dose per antigen and injected intramuscularly (50 μl each in both thighs). Serum samples were analyzed for antigen specific total immunoglobulin IgG and HI titers, details in^5^.

### Assessment of cellular responses

To study cellular responses, a similar study to the one described above was performed with 0.1 µg monovalent vaccine (A/Brisbane/59/2007). While the dosing regimen remained the same as the previous study, T-cell responses were analyzed by collecting spleens at 4 weeks post 2^nd^ immunization (4wp2). Single cell suspension from the spleens was prepared and the cells were stimulated with 0.1 µg of A/Brisbane/59/2007^6^.

Cultures were incubated overnight at 37 °C and then cells were stained for CD4 (Pacific Blue, BD Biosciences #558107, Clone RM4-5) and CD8 (Alexa Fluor700, BD Biosciences #557959, Clone 53–6.7) population, fixed with Cytofix/Cytoperm (BD Biosciences #554722), and then stained for intracellular cytokines. To stain intracellular cytokines peridinin-chlorophyll protein (PerCP) Cy5.5 (eBioscience #45-7311-82, Clone XMG1.2) was used for Interferon gamma (IFN-γ), BV605 (BioLegend #503829, Clone JES6-5H4) for interleukin-2 (IL-2), APC (BioLegend #504306, Clone TRFK5) for interleukin-5 (IL-5), PE (eBioscience #12-7133, Clone eBio13A) for interleukin-13 (IL-13) and Alexa Fluor488 (BD Biosciences #557719, Clone MP6-XT22) for tumor necrosis factor alpha (TNF-α) were used. These cells were analyzed on BD LSRII using appropriate voltage controls and compensation controls generated by mixing BD CompBeads and the above-mentioned dyes. Gating on CD4+ CD8− cells, the different T-helper subsets were identified as follows: IL-2+ and/or TNF-α+, but negative for IFN-γ or IL-5/IL-13 (Th0), as before but positive for IFN-γ (Th1), as before but positive for IL-5 and/or IL-13 (Th2).

### Immune cell recruitment studies at SOI and dLN

Ovalbumin Alexa Fluor 647 conjugate (OVA-AF647) from Thermo Scientific (catalog # O34784) was used for cell recruitment studies. GentleMACS Dissociator from Miltenyi Biotec was used along with the Skeletal Muscle Dissociation Kit (130-098-305) to prepare single cell suspension of muscles. PBS and HBSS medium was obtained from GIBCO and Collagenase D and DNase I were procured from Roche. For flow cytometry, cells were stained with Live/Dead Fixable Yellow Dead Cell Stain kit (Invitrogen) and combinations of the following antibodies: α-Ly6C-FITC, α-Ly6G-PE, α-CD11b-PE-Cy7, α-CD3-PerCpCy5.5 (all from BD Pharmingen) and α-I-A/I-E-Alexa-Fluor700, α-F4/80-PacificBlue, α-CD11c-APC-eFluor780 (all from eBioscience). Mice were divided into the following experimental groups: naïve, vaccinated with non-adjuvanted OVA-AF647 antigen or OVA-AF647 mixed 1:1 v/v with SEA20, MFA160 or SEA160 respectively. Each mouse was administered 50 μl bilaterally into the quadriceps. Three mice per group were euthanized at 6 h and 72 h, and six mice per group were euthanized at 24 h and 48 h. The quadriceps muscles and dLN were harvested for all mice. Single cells were isolated from muscles using skeletal muscle dissociation kit according to the manufacturer’s instructions. Single cell isolation from dLN was done as described before^7^.

### Statistical analysis

To assess humoral responses, statistical significance was tested by One-way ANOVA with post hoc analysis by Kruskall Wallis’s multiple comparison using non-adjuvanted vaccine (PBS + TIV) as a control. One-way ANOVA with post hoc analysis by Dunnett’s multiple comparison was used to compare humoral responses of SEA160 and MF59. Statistics for the immune cell recruitment studies are mentioned in the supplemental section.

## Results

The focus of this work was to further challenge the previous preliminary observation that larger emulsion droplets (160 nm) were more potent than small ones (20 nm and 90 nm), and to understand why. We evaluated whether droplet size alone was sufficient to result in different immunological outcomes, or if other biophysical attributes (eg. oil:surfactant ratio), also contributed. We evaluated formulations of the same composition but different droplet sizes, alongside formulations of the same droplet size but varying composition.

### Screening of squalene, Span85 and Tween80 to self-emulsify at 160 nm

Using the screening approach described in Shah *et al*., five different ratios of oil and surfactants were mixed overnight at room temperature (RT), and emulsified the next day with deionized water at a ratio of 1:20 v/v^5^. These mixtures were heated at 40 °C for 1 h and sized on Malver Zetasizer by Dynamic Light Scattering (DLS). Emulsion 4 from Table 1 was selected based on its droplet size, and was re-formulated by adding the oil to 10 mM citrate buffer, pH 6.5 heated at 40 °C. This primary emulsion was then heated at 40 °C for 1 h and then sized on DLS after 1:100 dilutions with deionized water. This formulation once optimized gave 160 nm droplet size and was termed as Self-Emulsifying Adjuvant 160 (SEA160).

**Table 1:** Screen of excipients to formulate SEA <200 nm.

Number	Squalene (%v/v)	Span85 (%v/v)	Tween80 (%v/v)	Dilution with aqueous phase	Z-avg (nm)	PDI
1	50	10	40	20	66.07	0.16
2	60	10	30	20	89.48	0.144
3	70	10	20	20	84.45	0.161
4	70	15	15	20	188	0.188
5	70	20	10	20	2798	0.877

Squalene, Span85 and Tween80 were mixed overnight and diluted with deionized water next day. Each mixture was heated at 40 °C for 1 h. Emulsions were diluted 1:100 with deionized water and sized for particle size and polydispersity index (PDI) on Malvern Zetasizer. Emulsion # 4 was further optimized to SEA160.

### Physical characterization of SEA160

Size of SEA160 was 160 nm with a very low PDI indicating a homogenous formulation; Table 2 shows results from three independent formulations. The concentration of squalene in SEA160 is lower than MF59 and different from the control emulsions that were used for *in vivo* comparison in order to assess differential impact of droplet size, oil content or surfactant ratio; Table 3 gives a comprehensive overview of formulations with respective similarities and differences in (i) size, (ii) squalene oil content and (iii) surfactant ratios. Figure S1 shows the transmission electron microscopy image of SEA160 with a scale bar of 100 nm.

**Table 2:** Physical characterization of sterile filtered SEA160.

Z-avg (nm)	PDI	Zeta Potential (mV)	Osmolality (mOsm/kg)	pH	Squalene (mg/ml)
157.47 ± 2.8	0.093 ± 0.02	−54.2 ± 1.93	33.66 ± 1.15	6.69 ± 0.04	28.98 ± 0.22

SEA160 post filtering through a 0.22 µm membrane filter was analyzed for droplet size, zeta potential, pH, osmolality and squalene content. Values are expressed as average ± standard deviation, n = 3.

**Table 3:** Control emulsions for *in vivo* comparison of impact of droplet size (Formulations of same composition but different droplet size).

Formulation	Adjuvant Effect	Droplet Size (nm)	Composition (%v/v)		
			Squalene	Span85	Tween80
SEA20	+	**20**	1.49	0.49	3.48
MFA90	++	**90**	1.49	0.49	3.48
MFA160	+++	**160**	**1.49**	***0.49***	***3.48***
SEA160 (diluted)	**++++**	**160**	**1.49**	***0.32***	***0.32***
SEA160	**++++**	160	**3.5**	0.75	0.75
MF59	**++++**	160	**4.3**	0.5	0.5

### Humoral responses to differentially adjuvanted TIV antigens by ELISA and HI titers

We used various emulsions to compare the potency of formulations with the same composition, but different droplet sizes (SEA20, MFA90, MFA160). In addition, we assessed the impact of oil content by comparing formulations of the same size, with increasing oil content (MFA160, SEA160, MF59), and we also evaluated the impact of different oil/surfactant ratio^5^ (Table 3).

**Figure 1:**
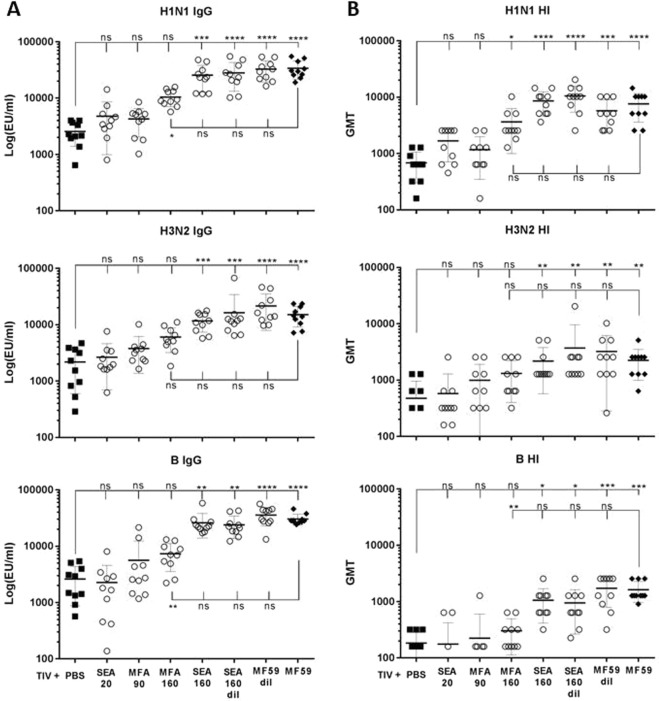
Antibodies against influenza antigen induced by vaccination with different adjuvants. Trivalent inactivated influenza virus antigens: H1N1 A/California/7/09, H3N2 A/Texas/50/2012 and B/Massachusetts/2/2012 were administered twice at 0.1 µg three weeks apart. Animals were divided into 10 animals per group and were administered PBS, non-adjuvanted TIV and TIV adjuvanted with SEA20, MFA90, MFA160, SEA160, MF59, diluted SEA160 and diluted MF59. Sera from 2wp2 were analyzed by ELISA (**A**) to assess IgG for each antigen individually. One-way ANOVA with post hoc analysis by Dunnett’s multiple comparison using MF59 for comparative purposes showed that adjuvants having 160 nm droplet size, especially SEA160, diluted SEA160 and diluted MF59 were not statistically different than MF59. One-way ANOVA with post hoc analysis Kruskall Wallis’s multiple comparison using PBS for comparative purposes showed that 160 nm adjuvanted groups - SEA160, diluted SEA160, MF59 and diluted MF59 were statistically higher than PBS. (**B**) Functional hemagglutination inhibition (HI) titers were analyzed for each antigen individually. One-way ANOVA with post hoc analysis by Dunnett’s multiple comparison using MF59 for comparative purposes showed that for H1N1 and B/Massachusetts adjuvants with droplet size of 160 nm, especially SEA160, diluted SEA160 and diluted MF59 were not statistically different than MF59. One-way ANOVA with post hoc analysis Kruskall Wallis’s multiple comparison using PBS for comparative purposes showed that for H1N1 and H3N2 antigens 160 nm adjuvanted groups - SEA160, diluted SEA160, MF59 and diluted MF59 were statistically higher than PBS.

As described previously for a set of different antigens and mouse strains^5^, the greater the droplet sizes of the emulsions, the greater the adjuvant potency, as defined by increased IgG titers against co-formulated antigens: SEA20 <MFA90 <MFA160 (Fig. 1). Moreover, adjuvant potency was also impacted by emulsion composition, since SEA160 and MF59 (160 nm), also induced higher titers than MFA160 (160 nm) (MFA160< SEA160 = MF59), because SEA160 and MF59 have higher squalene content than MFA160. To determine if oil content and/or droplet number impacted the adjuvant effect, we diluted both SEA160 and MF59 to an equivalent squalene content as MFA160. But both diluted formulations achieved similar titers as their non-diluted counterparts, pointing to the fact that individual droplet composition appeared to have an impact on potency, and not the oil-content or droplet number. SEA160 and MF59 have an equal ratio of surfactants Span85 and Tween80, while the surfactant ratios of SEA20, MFA90 or MFA160 are different (high Tween80; low Span85) (Table 3). Only SEA160 and MF59 (diluted or non-diluted) induced statistically higher IgG titers compared to non-adjuvanted antigens (Fig. 1A), while the titers induced by SEA160 were not statistically different to MF59. For more exhaustive statistical analysis of the data set refer to Supplemental Table 1 A and B.

The sera were also analyzed for hemagglutinin inhibiton titers to assess functionality of antibodies (Fig. 1B**)** with similar results. Importantly, these findings were also confirmed for a higher dose (1 µg) of antigens or for a monovalent influenza vaccine (Supplementary Figs S2, S3).

### Assessment of cellular responses

**Figure 2:**
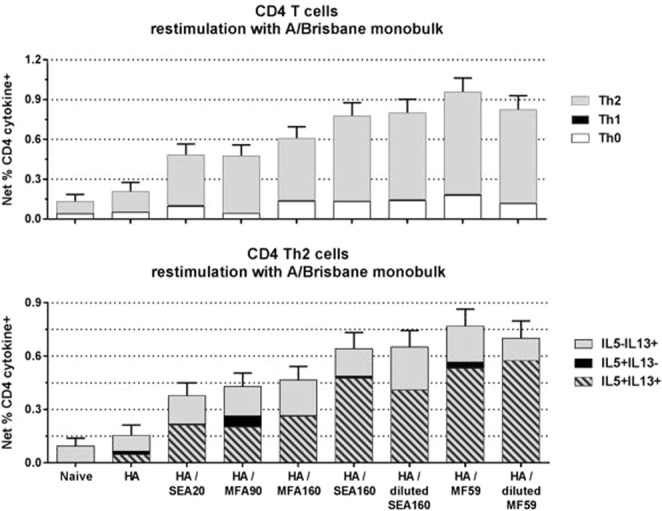
T cell responses assessed for flu antigen adjuvanted with novel emulsions Balb/c mice at 4wp2. Spleens were harvested from the treated animals and single cell suspension was generated. CD4+ positive T cells were re-stimulated *in vitro* with the protein used for immunization and were analysed by flow cytometry. T-helper subsets (Th0, Th1 or Th2) were identified as described in Material and Methods.

To evaluate the cellular immune responses induced by the different formulations, animals were euthanized at four weeks post second immunization (4wp2) and T cells isolated from spleens were re-stimulated *in vitro* with vaccine antigens and analyzed for intracellular cytokine staining by flow cytometry. Similar to the humoral responses, we observed a size- and composition-dependent trend for the T-helper cell responses (SEA20 < MFA90 < MFA160 < SEA160 = MF59) (Fig. 2). SEA160 and the commercial benchmark MF59 generated a similar profile in terms of quantity and quality of activated cytokine secreting cells. As previously shown for MF59 in Balb/C mice, the vaccines exhibit a Th2-biased profile as characterized by both IL-5 and IL-13 producing cells^6^. Also, sera from these animals were collected at 2wp2 and analyzed for IgG responses by ELISA with similar results to the previous flu studies (Supplementary Fig. S2).

### Immune cell recruitment studies

**Figure 3:**
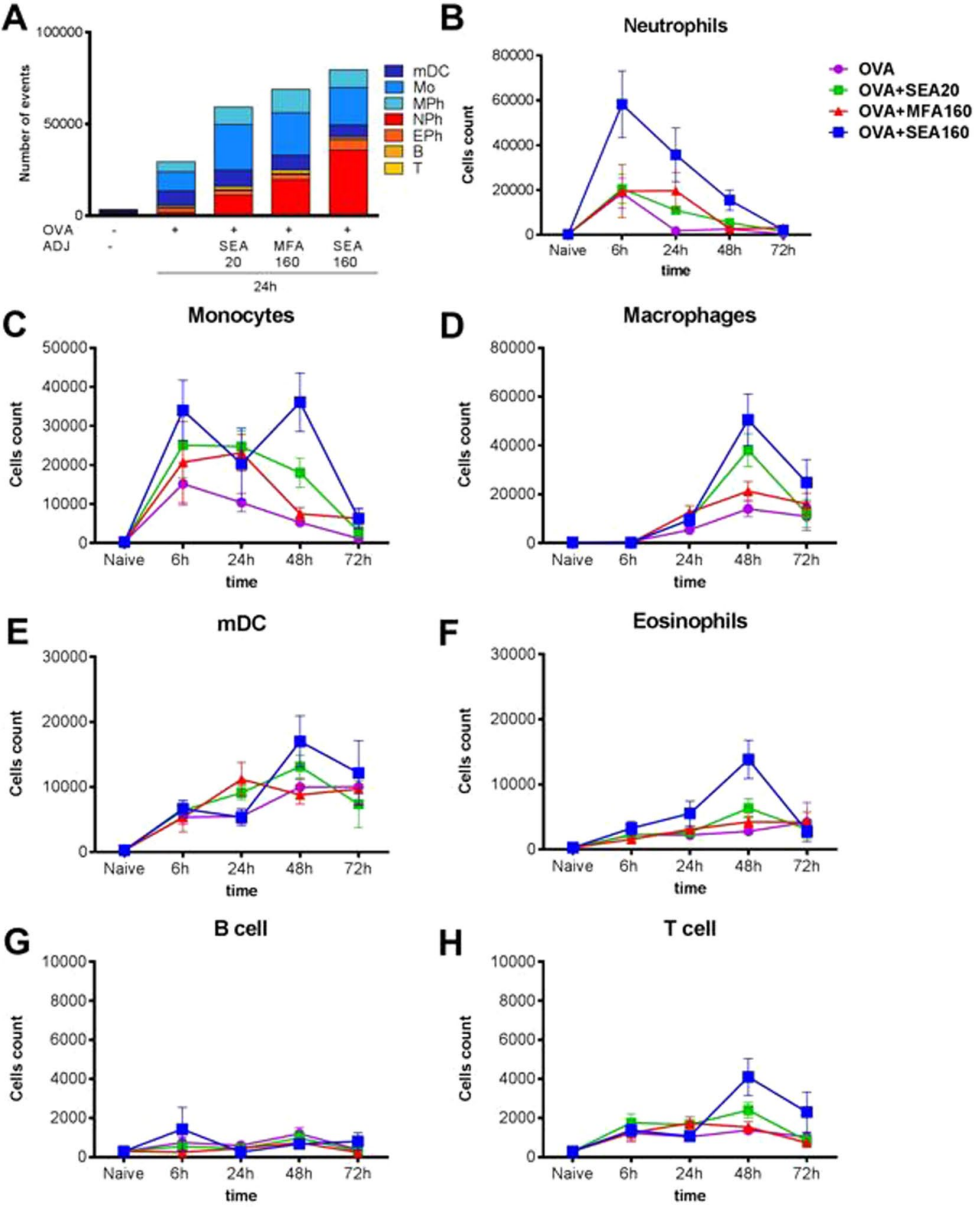
Cell recruitment induced by selected adjuvants at different timepoints post-vaccination. Mice were immunized IM with fluorescent OVA-AF647 together with the adjuvants of interest. At various timepoints animals were euthanized and organs harvested for analysis of immune cell composition at SOI. Once the muscle cells were homogenized into a single cell suspension and stained with the antibody mixture, they were analyzed by multi-color flow cytometry. Number of animals per time-point were: 3 animals for 6 h and 72 h, and 6 animals for 24 h and 48 h.

**Figure 4:**
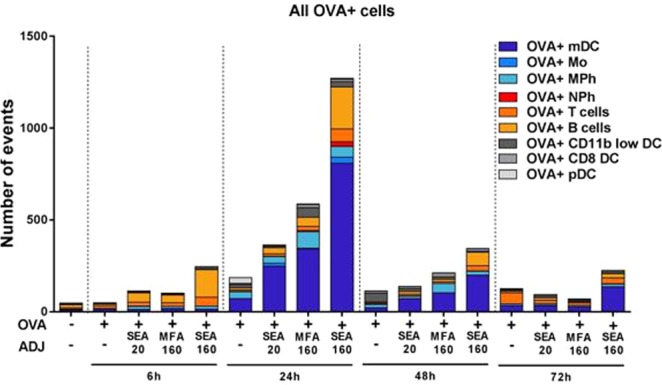
Antigen positive immune cells at the dLNs of same mice shown in Fig. 3. Once the dLNs were homogenized into a single cell suspension and stained with the antibody mixture, they were analyzed by flow cytometry. Numbers of animals per time-point were: 3 animals for 6 h and 72 h, and 6 animals for 24 h and 48 h. Each animal was  injected in both thighs; so each animal indicates two replicates since dLNs near both SOI were harvested.

Efficient activation of adaptive immunity (antibody and T cell responses) is preceded by effective stimulation of innate immune sensors. It is widely believed that vaccine adjuvants mediate their action via these innate signaling cascades. Hence, we analyzed the quantity and quality of immune activation by the different formulations directly at the SOI (Fig. 3) and at dLN (Fig. 4). Since we wanted to study the effect of droplet size and emulsion composition on immune response, we selected SEA20, MFA160 and SEA160 for this study. Specifically, we measured numbers and cell types of cellular infiltrate in the SOI and followed uptake and translocation of fluorescently labeled antigen by these cells from SOI to dLN.

As shown in Fig. 3A, while there were very few immune cells in muscles from naïve animals, all emulsions activated innate immunity efficiently, and induced recruitment of cells from the blood stream to the SOI. As described for MF59, cell recruitment started within 1 h, peaked within 48 h and for most emulsions, local effects were resolved after 72 h^7^. Again, emulsions with a greater droplet size (MFA160 and SEA160) induced greater cell recruitment than non-adjuvanted or smaller-sized emulsions. Also, optimized droplet composition (SEA160) further enhanced this trend, which correlated with the earlier data from the humoral immune responses.

Figure 3B–H shows cellular recruitment kinetics over 3-days post-injection. Pro-inflammatory cell types (Neutrophils/NPh and Ly6c+ monocytes/Mo) were the first to be recruited and were the most abundant cell types. Again, SEA160 induced the highest recruitment of these cells. Monocytes revealed a peculiar bi-phasic recruitment pattern that was observed in two independent experiments with six individual muscles each.

As shown for MF59, the pro-inflammatory cells are closely followed by myeloid dendritic cells/mDCs and macrophages/MPh and eosinophils/EPh as a second line of defense. Consistent, but lower recruitment was observed for adaptive immune cells – B and T cells (statistics in Supplemental Tables 2, 3).

Cell infiltration into the SOI precedes antigen uptake and translocation to dLNs. So, the dLN from the same mice were also analyzed by flow cytometry to study the number of antigen positive immune cells over time. While the muscles were analyzed for infiltrating immune cells (with or without antigen), in the dLN we focused exclusively on antigen-positive immune cells. Figure 4 consolidates the entire data set into one figure with all the groups and the time-points studied (statistics in Supplemental Table 4). We observed that SEA160, with greater droplet size and optimized oil/surfactant composition, at 24 h had the highest amount of antigen positive immune cells at the dLN, with the majority comprising of mDCs, which are the key APCs.

Since Ly6c+ monocytes differentiate into mDCs, it can be speculated that early recruitment of monocytes to SOI, where they eventually take up antigen and migrate to dLNs, accounted for the higher numbers of antigen positive mDCs. Also, it might explain the low population of monocytes observed for the SEA160 group at SOI at 24 h. Efficient antigen uptake and presentation in dLNs in response to adjuvant-stimulation accounts for activation of adaptive immune T- and B cells and hence effector function. Overall, in all readouts we observed the impact on potency achieved by emulsions of varying size and composition as follows: SEA20 < MFA160 < SEA160 = MF59.

## Discussion

In this work, we thoroughly explored the impact of emulsion droplet size and composition on the potency of squalene based oil in water emulsion adjuvants. While we had previously shown that smaller droplet size generated lower immunogenicity for co-administered model antigens^5^, here we substantiated that observation in a more comprehensive study and tried to understand why droplet size had such a significant impact. SEA160 was shown to be a highly effective adjuvant, which was produced in a simple process, without the need for complex equipment such as a microfluidizer. SEA160 had reproducible size, a low poly-dispersity index and potent adjuvanticity. In contrast, SEA20 was an inferior adjuvant compared to SEA160, primarily due to the difference in droplet size. This was a significant result that highlights that there are no benefits in reducing emulsion droplet size below the established size of ~160 nm.

Using TIV antigens we assessed the potency of the novel emulsion adjuvants in comparison to the established benchmark, the MF59 oil-in-water squalene emulsion that is included in licensed products. We established statistical equivalence of the novel SEA160 to MF59 and interestingly, confirmed an emulsion droplet size-dependent adjuvant impact, with 160 nm >90 nm >20 nm^5^. Further we can use the data generated here to build a rational hypothesis that effective immune activation by emulsion adjuvants is potentially mediated by the presence of surfactants on droplets, and that in addition to droplet size, surfactant can also play a major role in potency. Adjuvants enhance immune responses primarily via activation of innate immunity^7–10^. It was previously established for MF59 that the mix of the surfactants could activate innate immune cells, but that they were not sufficient, and only the fully formulated emulsion acted as an effective vaccine adjuvant^11^. Since it has been shown previously that emulsion droplets are internalized by host cells *in vitro* and *in vivo*^12^, efficient immune activation may require internalization of emulsion droplets into target cells, allowing access for the surfactants to an intracellular target. Adjuvant-mediated innate immune activation via release of endogenous danger signals (danger associated molecular patterns, DAMPs) such as ATP, host DNA or uric acid has been described and likely plays a part in adjuvanticity for all particulate adjuvants^13^. Also the modulation of intracellular compartments and activation of the endoplasmatic reticulum stress sensor and subsequent metabolic changes have been observed for the emulsion adjuvant AS03^14^.

Accumulating evidence shows that emulsions - and other particulate adjuvants, including alum, target myeloid cells, including monocyte/macrophages, neutrophils and dendritic cells^7,9,10,14,15^. We observed that SEA160 resulted in the highest infiltration of immune cells at the SOI, and the most rapid and efficient translocation of antigen-loaded immune cells to dLN. While the overall cell infiltration observed clearly reflects the relative differences in adjuvant potency, distinct differences also emerge from analyzing the respective cell compositions. Key players at the SOI would be potential antigen-presenting cells (APC), e.g. mDCs, monocytes and macrophages. These cells can take up antigen and transport it to dLN. But no differences in cell numbers were found for mDCs for the different formulations. However, differences for recruitment of inflammatory Ly6c+ monocytes that differentiate into mDCs were observed. This fuels speculation on whether the drop in numbers of monocytes at 24 h post-injection accounts for increased monocyte-to-DC differentiation, which was previously described for MF59^9,16^. Accordingly, the higher numbers of antigen-positive mDCs found at the lymph node at the respective time-points might represent these newly differentiated DCs.

Beyond the key observations made here, we believe that additional mechanistic studies are needed to answer key questions, including whether emulsion adjuvant internalization into target cells is a requirement for the adjuvant effect. Other interesting questions would be whether adjuvant size is sensed via a specific unknown receptor, and whether surfactants act on cellular sensors or indirectly via membrane perturbation. A major achievement described here is the development of the novel, and highly effective emulsion adjuvant SEA160. We developed this emulsion adjuvant using a simple and reproducible process which involves no expensive equipment, and we show that SEA160 induces immune responses with flu antigens comparable to the established MF59. In the bigger picture, we have shown that emulsion droplet size is an important parameter for adjuvant potency, and contrary to many suggestions, smaller is not ‘better’^4^. Further work is warranted to extensively explore and determine whether this is a common observation for particulate adjuvants and the need to create smaller particulates should be challenged further.

## Supplementary information

Supplementary Info

## Data Availability

Study data will be made available to readers upon request to the corresponding author.
