# Supplementary material for: The droplet size of emulsion adjuvants has significant impact on their potency, due to differences in immune cell-recruitment and -activation
Source: Sci Rep. 2019 Aug 8;9:11520. doi: 10.1038/s41598-019-47885-z (PMC6687744; doi:10.1038/s41598-019-47885-z)
Supplement: Supplementary file 1 — Supplementary Info [file 41598_2019_47885_MOESM1_ESM.docx]

**Supplementary Material**

**The droplet size of emulsion adjuvants has significant impact on their potency, due to differences in immune cell-recruitment and -activation**

Ruchi R. Shah^1,2,$^, Marianna Taccone^1^, Elisabetta Monaci^1^, Luis A. Brito^2$^, Alessandra Bonci^1^, Derek T. O’Hagan^2,3^, Mansoor M. Amiji^4^ and Anja Seubert^1*^

^1^ GSK, Siena, Italy

^2^ GSK, Cambridge, MA, USA

^3^ GSK, Rockville, MD, USA

^4^ Northeastern University, Boston, MA, USA

^$^ current address: Moderna Therapeutics, Cambridge, MA, USA, 02139

**Address correspondence and reprint request to**:

Anja Seubert, GSK, Via Fiorentina 1, Siena, 53100, Italy.

E-mail address: [anja.k.seubert@gsk.com](mailto:anja.k.seubert@gsk.com); phone +39 0577 53 9329


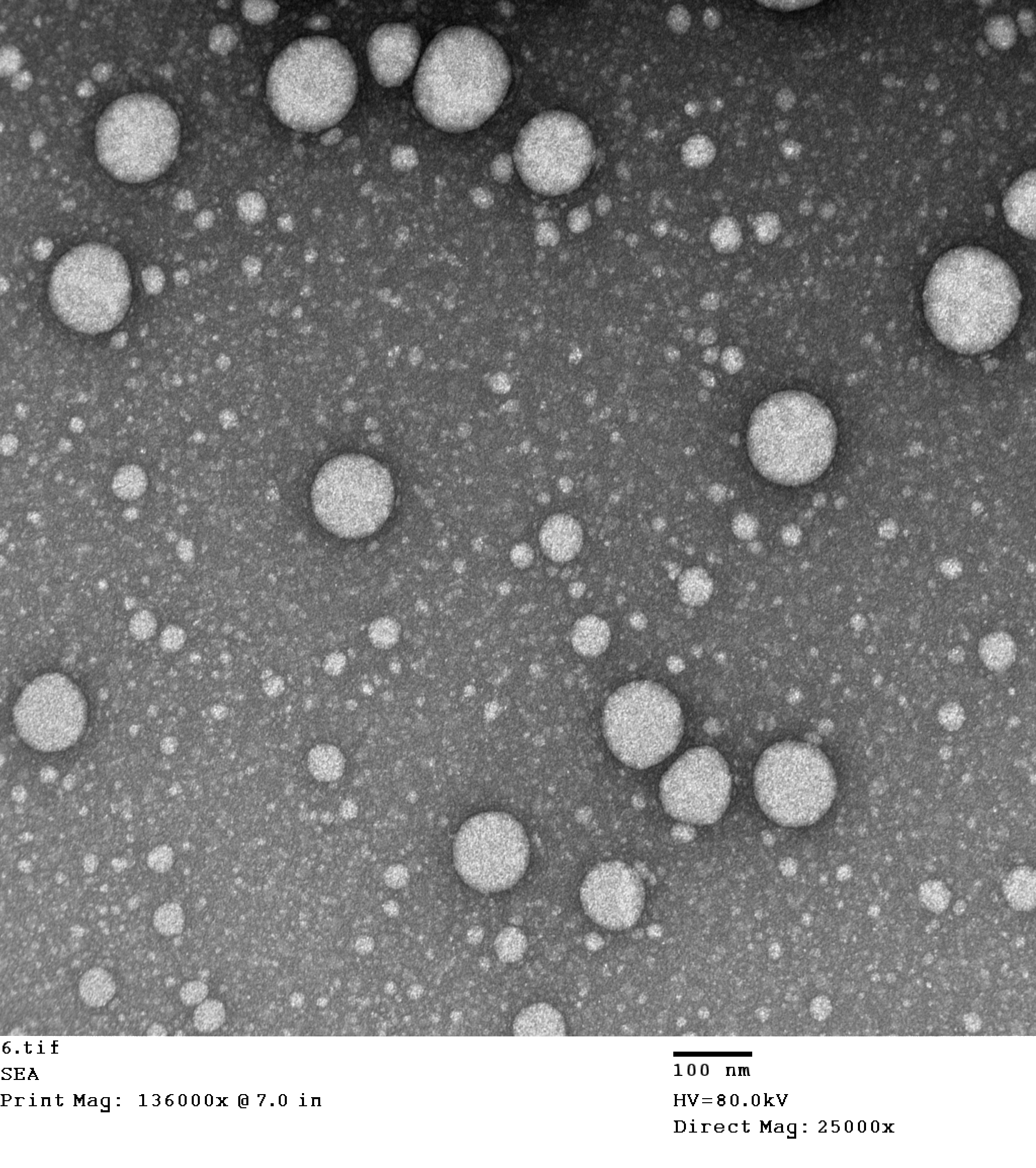


**S1: TEM image of SEA160:**

SEA160 was diluted with deionized water and stained with 1% uranyl acetate before imaging; scale bar is at 100nm.

**
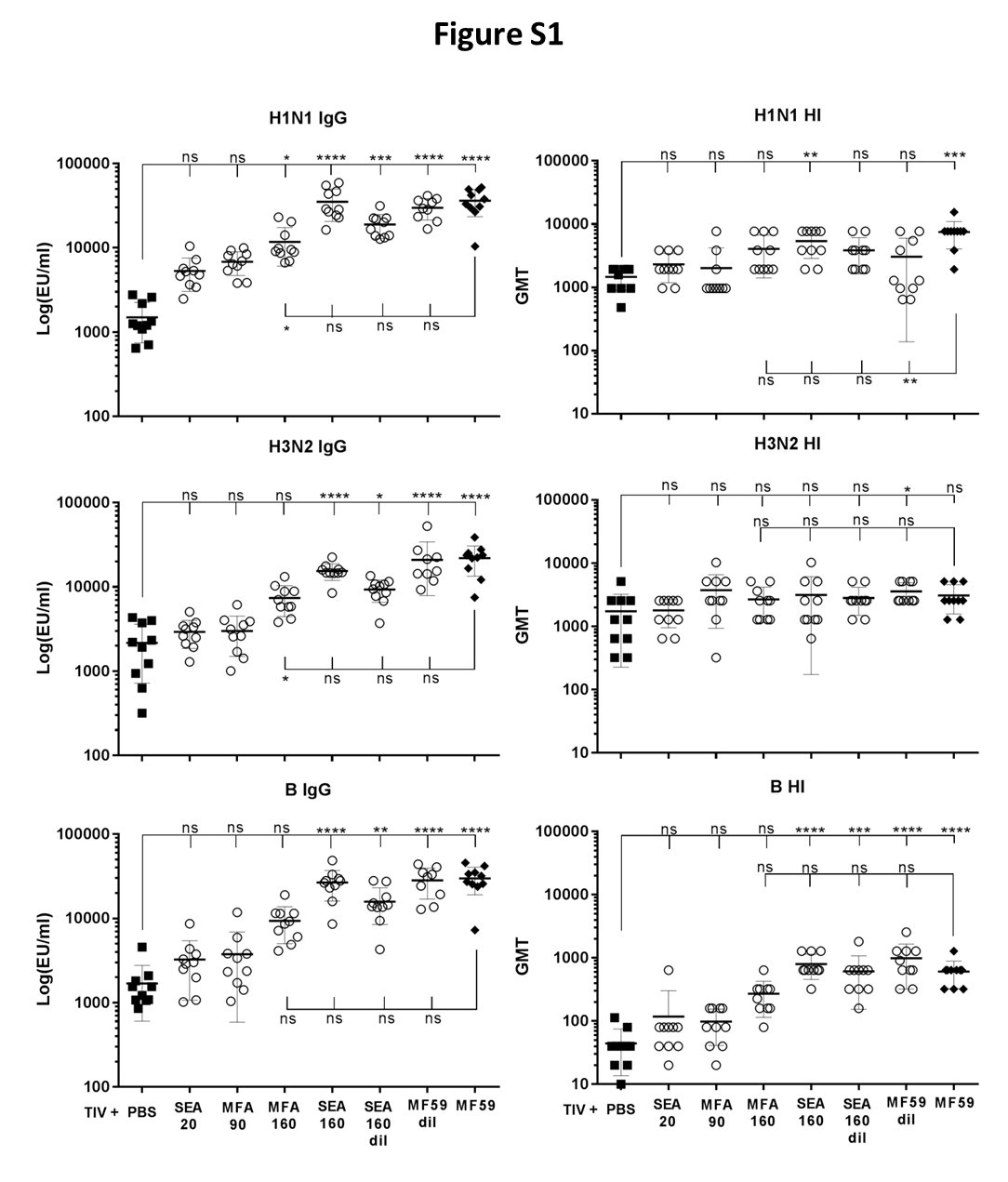
**

**S2(left panel): ELISA titers measuring adjuvanted responses using trivalent inactivated influenza vaccine antigens at 1µg dose each**

H1N1 A/California/7/09, H3N2 A/Texas/50/2012 and B/Massachusetts/2/2012 were administered twice at 1µg three weeks apart. Animals were divided into 10 animals per group and were administered PBS, non-adjuvanted TIV and TIV adjuvanted with SEA20, MFA90, MFA160, SEA160, MF59, diluted SEA160 and diluted MF59. Sera from 2wp2 were analyzed for IgG for each antigen individually. One way ANOVA with post hoc analysis Kruskall Wallis’s multiple comparison using PBS for comparative purposes showed that for H1N1 and B/Massachusetts 160nm adjuvanted groups - SEA160, diluted SEA160, MF59 and diluted MF59 were statistically higher than PBS. With H3N2 antigen SEA160, diluted MF59 and MF59 were statistically higher than PBS. One way ANOVA with post hoc analysis by Dunnett’s multiple comparison using MF59 for comparative purposes showed that 160nm adjuvanted groups - SEA160 and diluted MF59 were not statistically different than MF59 for all the three antigens.

**S2(right panel): Hemaglutinin inhibition titers measuring adjuvanted responses using trivalent inactivated influenza vaccine antigens at 1µg dose each**

H1N1 A/California/7/09, H3N2 A/Texas/50/2012 and B/Massachusetts/2/2012 were administered twice at 1µg three weeks apart. Animals were divided into 10 animals per group and were administered PBS, non-adjuvanted TIV and TIV adjuvanted with SEA20, MFA90, MFA160, SEA160, MF59, diluted SEA160 and diluted MF59. Sera from 2wp2 were analyzed for HI titers for each antigen individually. One way ANOVA with post hoc analysis by Dunnett’s multiple comparison using MF59 for comparative purposes showed that for H1N1 antigen SEA160 was not statistically different than MF59. For H3N2 only non-adjuvanted PBS group was statistically lower than MF59 and for B/Massachusetts all 160nm adjuvanted groups were statistically equivalent to MF59. One way ANOVA with post hoc analysis Kruskall Wallis’s multiple comparison using PBS for comparative purposes showed that for H1N1 antigen 160nm adjuvanted groups - SEA160 and MF59 were statistically higher than PBS and for H3N2 antigen there was no statistical difference on comparison with PBS. For B/Massachusetts SEA160, diluted SEA160, MF59 and diluted MF59 were statistically higher than PBS.

**
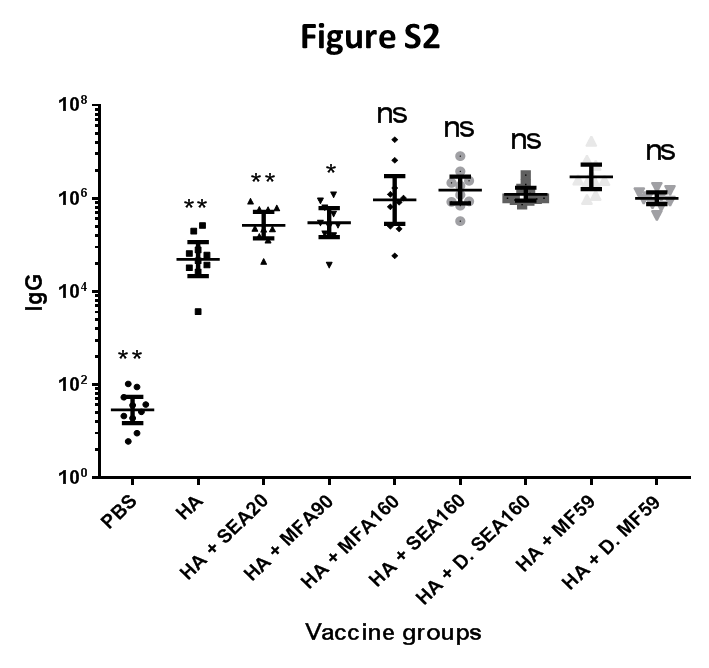
**

**Figure S3: ELISA titers measuring adjuvanted responses using H1N1 monobulk influenza vaccine antigen at 0.1µg dose**

Animals were divided into 10 animals per group and were adminsitered PBS, non-adjuvanted TIV and TIV adjuvanted with SEA20, MFA90, MFA160, SEA160, MF59, diluted SEA160 and diluted MF59. Sera from 2wp2 were analyzed for IgG. One way ANOVA with post hoc by Dunnett’s multiple comparison using MF59 for comparative purposes for 2wp2 sera showed statistically different result for PBS-control group, non-adjuvanted immunized mice and SEA20, MFA90 adjuvanted groups after the second immunization.

**Supplemental Table 1A**

| **Total IgG (2wp2) – Size Group** | **H1N1** | **H3N2** | **B** |
| --- | --- | --- | --- |
| PBS vs. SEA20 | ns | ns | ns |
| PBS vs. MFA90 | ns | ns | ns |
| PBS vs. MFA160 | **** | ** | ns |
| SEA20 vs. MFA90 | ns | ns | ns |
| SEA20 vs. MFA160 | * | * | * |
| MFA90 vs. MFA160 | * | ns | ns |

**Supplemental Table 1B**

| **Total IgG (2wp2) – Composition Group** | **H1N1** | **H3N2** | **B** |
| --- | --- | --- | --- |
| MFA160 vs. SEA160 | ** | * | *** |
| MFA160 vs. diluted SEA160 | ** | ** | ** |
| SEA160 vs. diluted SEA160 | ns | ns | ns |

**Supplemental Table 1 A + B: Detailed Statistics of data shown in Figure 1**

Sera from 2wp2 were analyzed by ELISA (A) to assess IgG for each antigen individually. Statistical analysis across the complete data set using PBS as comparator to assess adjuvant effect and MF59 as comparator for a commercial benchmark are depicted in Figure 1.

In order to address the impact of droplet size on adjuvant effect, a subset of data (PBS, SEA20, MFA90, MFA160) was analysed by One-way ANOVA with post hoc analysis Kruskall Wallis’s multiple comparison (Table 1A). Only MFA160 with the highest droplet size of 160 nm has a significant adjuvant effect when compared to PBS, while the smaller sized emulsions MFA90 and SEA20 were significantly less potent when compared to MFA160. Similar findings had been observed in a prior publication.

Similarly the significant impact of different surfactant composition at same oil content (MFA160 versus diluted SEA160) and the lack of impact due to overall oil-content (SEA160 versus diluted SEA160) was addressed in a subset of data by One-way ANOVA with post hoc analysis Kruskall Wallis’s multiple comparison (Table 1B).

**
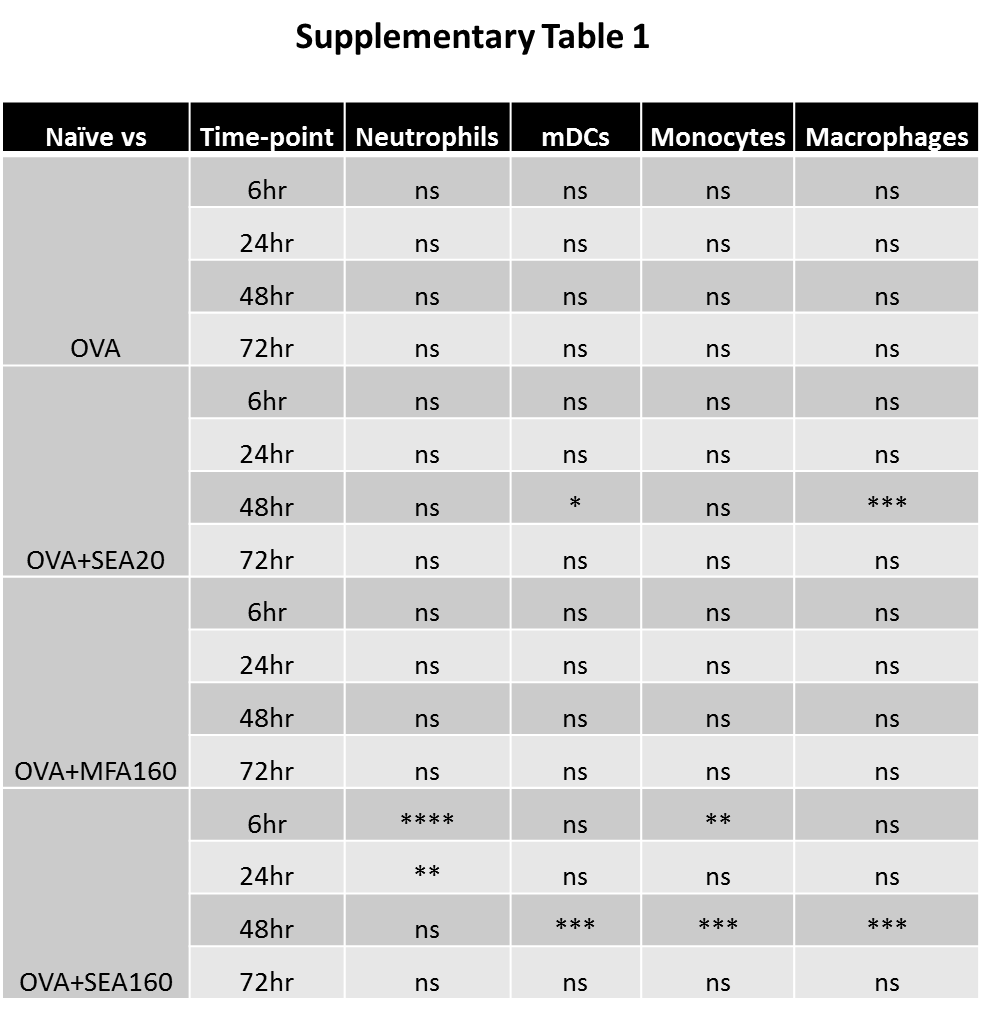
**

**Supplemental Table 2: Statistics for highly recruited APCs at SOI**

The naïve group of mice, untreated, from figure 3(A) were analyzed by one-way ANOVA using Tukey’s multiple comparison tests for the number of APCs at SOI at various timepoints. alpha = 0.05, ns = not significant, */**/***/**** = indicates level of statistical significance

**
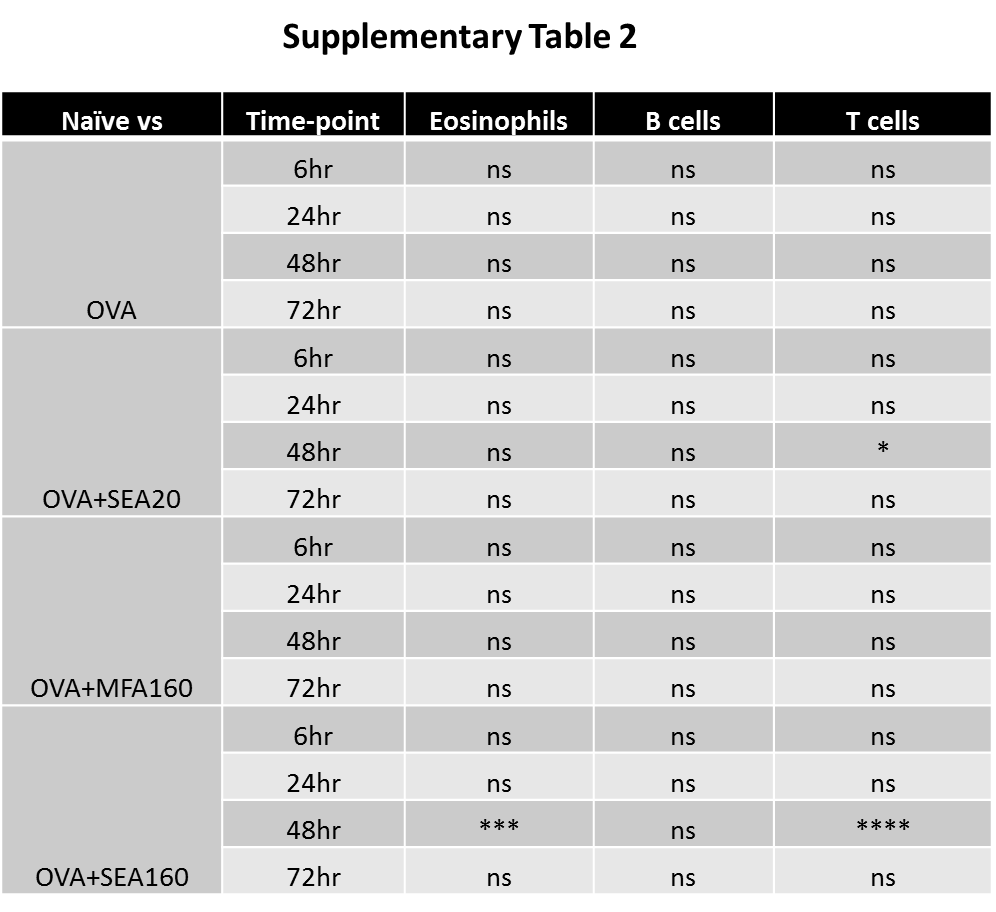
**

**Supplemental Table 3: Statistics for innate immune cells at SOI**

The naïve group of mice, untreated, from figure 3(A) were analyzed by one-way ANOVA using Tukey’s multiple comparison tests for innate immune cells at SOI at various timepoints. alpha = 0.05, ns = not significant, */**/***/**** = indicates level of statistical significance

**
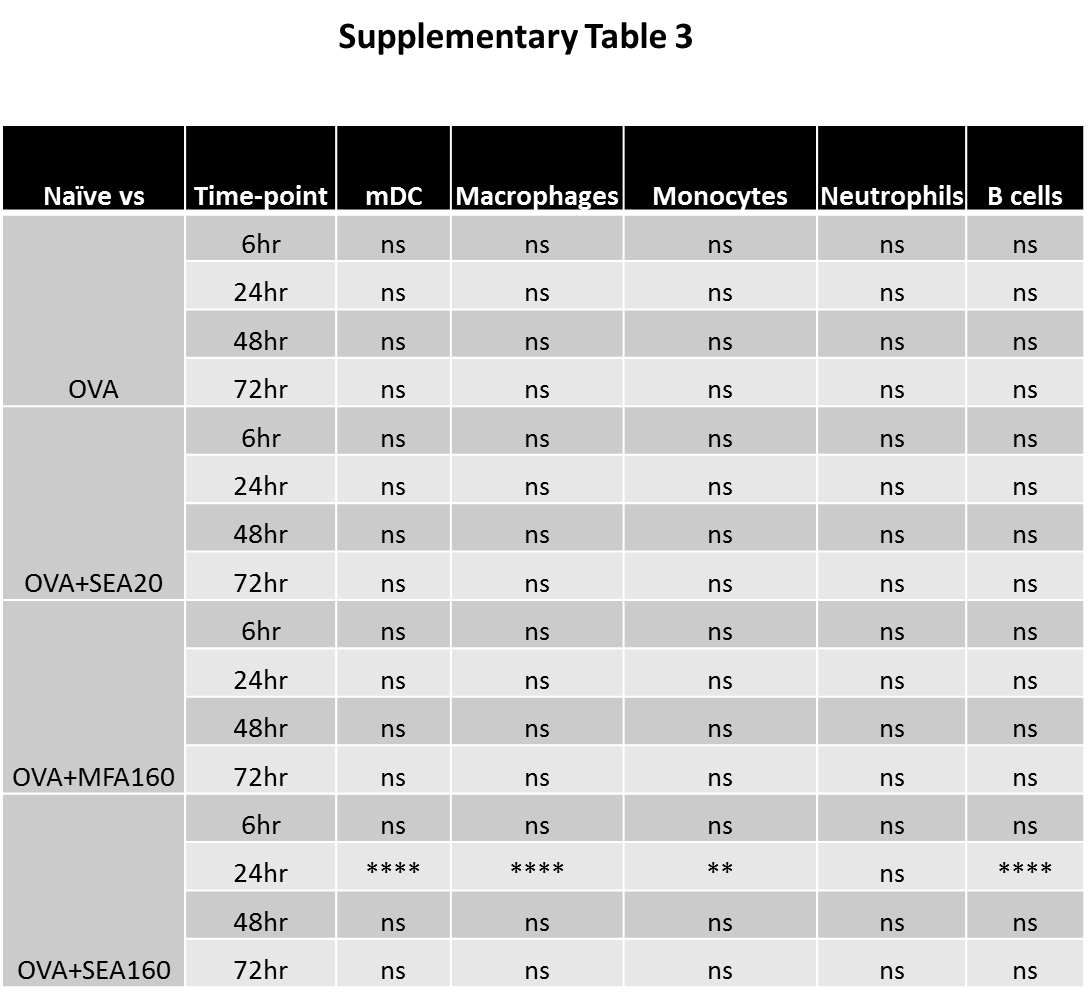
**

**Supplemental Table 4: Statistics for all the immune cells at the dLN**

One-way ANOVA using Tukey’s multiple comparison tests was run for all the immune cells at various timepoints present in the dLN in Figure 3(B). alpha = 0.05, ns = not significant, */**/***/**** = indicates level of statistical significance.
